# “We might be put into situations we are uncomfortable with, but not exactly told how to deal with them”: Health professional students' experiences questioning low‐value care practices during work‐integrated learning

**DOI:** 10.1002/ase.70054

**Published:** 2025-06-05

**Authors:** Melanie K. Farlie, Jessica Coventry, Jonathan Foo, Samantha Sevenhuysen, Brendan Shannon, Cylie M. Williams, Stephen Maloney, Kristie Matthews

**Affiliations:** ^1^ Department of Physiotherapy Monash University Frankston Victoria Australia; ^2^ Monash Centre for Scholarship in Health Professions Education Monash University Clayton Victoria Australia; ^3^ School of Primary and Allied Health Care Monash University Frankston Victoria Australia; ^4^ School of Nursing and Midwifery Monash University Frankston Victoria Australia; ^5^ Peninsula Health Frankston Victoria Australia; ^6^ Department of Paramedicine Monash University Frankston Victoria Australia

**Keywords:** health communication, learning, low value care, patient safety, professionalism, speaking up, work integrated learning

## Abstract

Health professions students often observe and practice alongside supervising health professionals during work‐integrated learning (WIL) to develop essential capabilities. While students may encounter practices they interpret as low‐value care during WIL, many hesitate to question or challenge these practices. This study aimed to (1) explore students' perceptions of low‐value care and their experiences discussing it during WIL and (2) identify training priorities for education programs to support students and educators in navigating these conversations. A descriptive qualitative study was conducted with health professions students who had completed at least one clinical placement and participated in online interviews. A team‐based framework approach was used to identify themes from the data. Thirty‐six students from 10 health professions (average age 26 years) were interviewed. Three themes were identified: (1) student conceptualizations of low‐value care are multi‐faceted, (2) students need safety to initiate conversations about perceived low‐value care on clinical placement, and (3) students seek practical training and clear guidance to handle complex conversations during placement. This study highlights the nuanced ways students perceive low‐value care, encompassing respect, compassion, and trust, in addition to clinical guidelines. It underscores the importance of pre‐placement training and the need to co‐design education programs involving students, educators, and healthcare consumers to improve communication skills around low‐value care. These findings offer actionable insights for developing supportive teaching and learning interventions.

## INTRODUCTION

A key feature in the education of health professional students is the incorporation of work‐integrated learning (WIL) into the course curricula. Work‐integrated learning is a pedagogical strategy that enables healthcare students to consolidate the application of theoretical knowledge into clinical practice.^1^ Students are commonly situated within a healthcare workplace, observing and practicing alongside supervising health professionals as they develop the capabilities necessary for their future qualifications. Work‐integrated learning experiences may occur for several weeks or months, with or without concurrent academic learning, enabling students to become acculturated to their chosen profession.^2^ It is essential to acknowledge the complexities of the healthcare organization as a learning environment and the many aspects that may influence the effectiveness of student learning within this context.^3^

Healthcare professional students undertaking WIL experiences in health services will likely observe clinical practice considered ‘low‐value’ are at some stage in their training. Care is considered ‘low‐value’ when the evidence for an intervention suggests it confers no or minimal benefit to healthcare consumers or the risk of harm exceeds any likely benefit.^4^ An Australian audit of 35,573 healthcare encounters identified that 43% of healthcare practice was inconsistent with current clinical guidelines.^5^ Although students may observe low‐value practice during WIL, they may be reluctant to question and challenge observed practices with supervising health professionals. This reluctance may be due to their subordinate position in the student–supervisor relationship, perceived potential or actual impacts on further learning opportunities, or fear of a negative impact on their summative high‐stakes assessment from their WIL supervisor.^6^ In addition to fearing repercussions from questioning supervisors, speaking up behaviors can also be influenced by knowing strategies how to speak up and anticipating a useful outcome from doing so.^7^, ^8^, ^9^ Student reluctance to speak up limits the opportunity to examine potential low‐value care delivery and why the clinician chose it. Having robust conversations with supervisors about low‐value care provides the opportunity to influence learning of the complexities of contemporary practice. Additionally, speaking up to sub‐optimal practices is vital to minimize the risk of harm to the healthcare consumer.^10^

Previous research has reported that student‐initiated conversations about practice observations during WIL can influence clinician practice.^6^ The study by Sevenhuysen et al.^6^ explored educators' perspectives (i.e., clinical educators, tutors, or preceptors) on health professional students' initiating conversations that challenged observed practice. A set of principles or perceived etiquette was identified for how educators thought students should approach potentially challenging conversations. Although study participants reflected that such conversations could feel confronting, many were able to identify when student questions led them to change their practice. This suggests that there may be benefits for educators and students engaging in conversations that challenge healthcare delivery.

This current study aimed to extend these earlier findings by exploring students' experiences initiating conversations about perceived observations of low‐value care during WIL and seeking students' perspectives on the educators' recommendations reported by Sevenhuysen et al.^6^ Integrating students' perspectives with those previously collected from educators can broaden our understanding of conversations challenging low‐value care in the WIL context. This may be valuable to guide education interventions for students and educators that build effective communication around contemporary practice. Therefore, this study aimed to (1) explore students' perceptions of low‐value care and their experiences discussing it during WIL and (2) identify training priorities for education programs to support students and educators in navigating these conversations, taking into account previously reported educator recommendations.

## METHODS

### Design

This descriptive qualitative study^11^ was situated within a social constructivist perspective.^12^ It used a framework analysis approach to conduct team‐based thematic analysis.^13^ The Monash University Human Research Ethics Committee granted ethical approval (Approval #35565).

### Participants

Potential participants were students studying entry‐to‐practice qualifications in any health profession (e.g., medicine, nursing, paramedicine, physiotherapy, and podiatry). To be eligible to participate, students must have participated in at least one WIL experience in a clinical setting, be studying at an Australian or Aotearoa New Zealand university, and be able to complete an online interview.

### Recruitment and consent

Health professional students were recruited using purposive sampling. Recruitment was conducted using multiple channels to maximize reach. This included social media posts (LinkedIn, Twitter/X, and Facebook), use of researcher networks and snowballing to request on‐campus advertising, learning management system forum board posts and email distribution lists at multiple health professional university programs across Australia and Aotearoa New Zealand. The method/s used at each university were determined by local policies and processes for advertising research opportunities to health professions students. The recruitment strategy for this study was an independent “opt‐in” activity not linked to participation in any curriculum related to the student's current health professional course. Interested students submitted an expression of interest collected and managed using the REDCap electronic data capture tool hosted and managed by Helix (Monash University).^14^, ^15^ All participants were presented with detailed information about the project and recorded consent via REDCap if willing to proceed. Students were offered a small monetary payment ($20 gift card) as compensation for their time participating in the study.

### Data collection

#### Demographics

All participants completed a demographic survey via REDCap at the time of consent. Participants recorded their age, gender, course discipline, course type, year of study, the number of WIL experiences completed, and the total number of weeks completed. Following the provision of demographic information, an online interview on Zoom (Zoom Video Communications, Inc., San Jose, CA) was organized.

#### Semi‐structured interviews

Interviews were conducted by one of five research team members (MF, JF, KM, BS and JC). Interviewers were registered health professionals with post‐qualification experience ranging from 2 to 25 years, with experience teaching and supervising health professional students in academic and clinical settings. Interviewers were allocated to eliminate any perceived or actual influence on participant assessment outcomes, training programs, or relationships with clinical placement providers. When a participant was studying the same profession as their interviewing team member, this was only allowed when the research team member was not faculty in the course or department that the student was enrolled in.

The lead researcher (MF) developed a semi‐structured interview guide, and the full research team provided three rounds of review to guide refinement before data collection commenced. Questions explored student conceptualizations of low‐value care, presented them with the educator recommendations from the Sevenhuysen et al.^6^ study to reflect on and respond to, and explored the students' perceptions of their training needs related to initiating conversations about observed low‐value care during WIL. The interview guide was modified after the first interview to explicitly ask students what recommendations they would give educators about participation in conversations with students challenging current practice. The interview guide is shown in Appendix S1a.

Zoom interviews were recorded, and audio data were auto‐transcribed using Otter.ai (Los Altos, CA). Two researchers (JC and MF) reviewed the transcripts for accuracy and de‐identified them. All interviewing team members reviewed recordings of each other's early interviews and debriefed as an interviewing team to ensure consistency in approach. All interviewers completed and shared post‐interview structured reflections (see example in Appendix S1b) and met regularly to discuss interview reflections throughout the data collection period.

### Information power

The principles of information power were used to determine when to cease recruitment and data collection.^16^ To judge the information power, the team considered (1) the narrow study aim: *student speaking up behavior and training needs in response to observing perceived low‐value care in the WIL context*, (2) the specificity of the sample: *health professions students who had experienced WIL*, (3) the application of theory: *social constructivism as applied to learning contexts*, (4) the richness of the dialogue: *in‐depth interviews conducted by interviewers from a range of professions with extensive experience working with students in WIL and academic contexts*, and (5) the cross‐case analysis strategy: *examination of cases describing varied perspectives and responses to the observation of perceived low value care*. Determination of information power included the consideration of interview data, post‐interview interviewer reflections, and shared decision making through team discussions. These judgments and processes enabled the team to determine when the collected data was sufficient to address the research aims.

### Team reflexivity

Project team members represented a diversity of clinical education, healthcare (physiotherapy, podiatry, paramedicine, and radiation therapy), qualitative research experience, and current positions in academia and health services. The members of this team shared extensive experience in the preparation of students for clinical placements at university (MF, SM, CW, JF, BS, and KM) delivery of curriculum on evidence‐informed practice (MF, CW, and BS) student supervision on clinical placement (MF, SM, CW, JF, SS, BS, and KM), investigating low‐value care and evidence translation (CW, SM, and JF), and management of student placement teams and clinical educators at the organizational level in health services (MF, SS, BS, and KM). A team‐based approach to reflexivity was integrated into this project to make team biases and assumptions explicit and to optimize teamwork during project planning, data collection, analysis, and interpretation.^17^ Approaching reflexivity as a team stimulated discussion on the team's shared orientation, assumptions and biases as a deliberate reflexivity practice throughout the project. Reflexivity statements from individual team members were collected using an online survey at three timepoints during the project. Team members reflected on prompts adapted from Barry et al.^17^ Individual reflexivity statements were then synthesized into a collective team reflexivity statement for the phase. Thematic syntheses were developed by three research team members (MF, KM, and JC) and then shared with the entire team for discussion and refinement during project team meetings. These discussions confirmed that the syntheses represented the team's orientation at each project stage. The thematic summaries identified five themes describing how the shared beliefs of the team may influence the interpretation and reporting of the study findings: *experience with student learning influences our perspectives; students are unlikely to raise their voices if they observe what they believe to be low‐value care; collecting student perspectives on low‐value care is critical to understanding how to support students during clinical education; analytical lenses* and *motivation to empower student voices drives our curiosity*. Syntheses of the team reflexivity statements at project initiation, pre‐recruitment, and data collection phases are presented in Appendix S2.

### Data analysis

**FIGURE 1:**
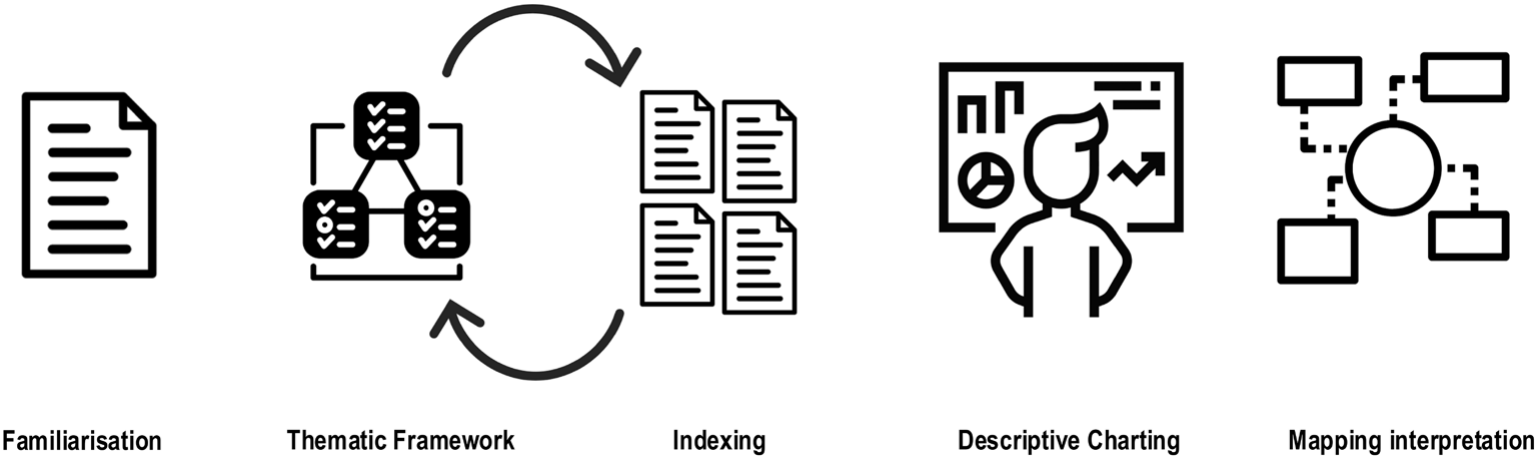
Visual representation of the five stages of framework analysis as described by Barry et al.^17^ adopted by the research team for data analysis and interpretation.

The framework analysis methods Ritchie and Spencer^13^ described were utilized to analyze the interview data. This approach to framework analysis consists of five stages: (1) familiarization, (2) thematic framework construction, (3) indexing, (4) charting, and (5) mapping and interpretation, as shown in Figure 1. The framework analysis method provided a staged approach that allowed all team members to contribute their perspectives and interpretations to the data analysis.

Data analysis was conducted using Microsoft Word 2019, Microsoft Excel 2019, and ATLAS.Ti 24. Analysis commenced with familiarization by reading and re‐reading transcripts, and then open, inductive coding was used to create the thematic framework over multiple coding rounds. Thematic framework development involved all team members who coded approximately half the interview transcripts between them. Each transcript was coded two to four times by different team members during this phase. Allocating transcripts to team members in this phase included consideration of allocation to team members who had and had not conducted the original interview and were not of the same discipline as the interview participant. These allocation decisions were made with the intention that many team perspectives contributed to the interpretation of the data and that all team members' perspectives contributed to the thematic framework. The transcript allocations for the thematic framework construction stage are shown in Appendix S3.

Coding rounds started with a team briefing and discussion to confirm the study research questions and coding process. This was followed by familiarization and independent coding. In addition to open coding, the first round required researchers to code data that highlighted participant values, attitudes, and beliefs and data showing conflict and cognitive dissonance to align with the first research question. This enabled the project lead (MF) to collate a preliminary thematic structure refined over the next coding round. After two rounds, the thematic framework was finalized, and the entire dataset was indexed by two researchers (JC and MF). Both coders noted challenges in applying the framework during independent indexing and met to discuss these challenges and review the data coded to each framework element. Where necessary, the code definition was adjusted to improve clarity and a shared interpretation of the data. No discrepancies between transcripts independently indexed by MF and JC were identified after the third transcript. Following indexing, the research team used descriptive charting to explore patterns across the dataset, and through exploration of the descriptive charting and multiple rounds of team discussion, the findings were mapped to themes aligned with the project's research questions. Of note, although student participants often referred to healthcare consumers as ‘patients’ or ‘clients’, the descriptions of findings have intentionally used the terms ‘healthcare consumer’ to be more inclusive.^18^

## RESULTS

### Participant demographics

Fifty‐three students consented to participate in the study. Of these, 36 were interviewed. The remaining 17 students either did not respond to interview schedule requests (*n* = 5), did not attend a scheduled interview nor respond to follow‐up invitations to reschedule (*n* = 8), or chose to withdraw from the study (*n* = 4). Participant characteristics are shown in Table 1.

**TABLE 1:** Study participant characteristics.

Characteristic	Participants (*n* = 36)
Age	
Range (years)	19–54
Average age—mean (SD)	26 (9.6)
Participants above 30 years	6
Participants below 30 years	30
Self‐identified gender	
Male/man	10
Female/woman	26
Non‐binary	0
Country and state	
*Australia*	34
Victoria	27
Queensland	3
South Australia	2
Western Australia	2
*Aotearoa New Zealand*	2
Number of placements	
Range	1–34
Average—median (IQR)	2 (2,4)
Total duration of clinical placements	
1–5 (weeks)	9
6–9 (weeks)	13
10–19 (weeks)	8
20+ (weeks)	4
Discipline of study	
Occupational therapy	5
Dietetics	3
Nursing and midwifery	2
Paramedicine	2
Pharmacy	5
Physiotherapy	5
Podiatry	1
Radiation therapy	3
Radiography	9
Social work	1

Participants were an average age of 26 years (range 19 to 54). Most participants self‐identified as female/woman, and most were from Victoria, Australia. The median number of placement experiences was two, ranging from 1 to 34. Most students had experienced less than 10 weeks of placement. However, 10% had experienced over 20 weeks. Students from nursing and midwifery and nine allied health professions participated. The highest proportion of students was in medical radiation practice (i.e. radiography and radiation therapy) (*n* = 12), with only single students studying podiatry or social work.

### Thematic results

Analysis identified three themes describing health professional student perceptions and experiences of managing conversations about low‐value care during WIL, and their training preferences to better enable such conversations. Students' *conceptualization of low‐value care was multi‐faceted*, which has implications for how to best teach effective response strategies to such observations and how educators might interpret this. Students also emphasized the *importance of safety and space in the WIL environment to enable conversations about low‐value* care without fearing consequences. Lastly, students determined that *educational interventions relating to low‐value care conversations must be practical, authentic, and applicable* across contexts.

#### Theme 1: Student conceptualizations of low‐value care are multi‐faceted

Students described various interpretations of what constitutes low‐value care (Table 2). They identified practices they perceived to lack respect, be ineffective or potentially harmful, or be enacted lazily or inadequately as low‐value. Students also noted the lack of continuity in care as low‐value care. They identified unnecessary interventions, including unnecessary exposure or redundant procedures, as examples of low‐value care, and they described them as not cost‐effective or evidence‐based. In contrast, other students described clinicians with poor attitudes toward work, completing inadequate assessments or working outside their professional scope of practice as delivering low‐value care. Students described low‐value care in the context of the care processes and healthcare consumer experiences, not clinical outcomes.

**TABLE 2:** Sub‐themes, properties of sub‐themes and indicative quotes for the theme “Student conceptualisations of low‐value care are multi‐faceted.”

Subtheme	Properties of sub‐theme	Indicative quote/s
Not respecting the healthcare consumer	Low‐value care is care delivery that does not incorporate the healthcare consumer's values and preferences. This could be exhibited by staff lack of compassion or kindness or insufficient or absent informed consent for procedures	“Her nurse came down to the X‐ray room [and] was like, ‘Oh, you now have to do the X‐ray’ …. And it seems like the patient didn't really know what was happening ….… she [the patient] was full‐on shouting … ‘I don't want to do this’ and all that. And then the nurse just sort of shouted back at her. So I don't think that's high‐value care at all … I know that nurse probably just wanted to get this procedure over and done with but then it's just not, it's not appropriate.” _[ID02_Radiog]_
Practice that is unnecessary, ineffective, and potentially harmful	Low‐value care is delivery that does not align with the healthcare consumer's needs and contemporary practice. This could be care not indicated by the healthcare consumer's condition, insufficient evidence for a procedure, or where the costs or risks outweigh the benefits	“What I would perceive as low‐value care would be initiating services, such as, for example, blood tests or other interventions, where the … risk of harm or the added costs would be outweighing the benefits that we are giving to the patients themselves.” _[ID49_Pharm]_
Inadequate attitudes or approaches to service delivery	Low‐value care is an action that does not support the continuity of care for the healthcare consumer. This could be inadequate handovers, lack of collaboration within the multi‐professional team, or providing incomplete assessments or insufficient explanations to the healthcare consumer	“Low‐value care in terms of physio is not completing an adequate assessment or providing adequate treatment for the patient. And I guess not getting the patient achieving the discharge goal they need to go home, for instance, sending them home without an adequate walking aid or … exercises.” _[ID07_PT]_

#### Theme 2: Students need safety to initiate conversations about perceived low‐value care on clinical placement

Students identified that low‐value care practices were observed while on placement, but their willingness to initiate conversations about these practices varied (Table 3). Students described a need to minimize negative consequences for themselves and healthcare consumers and for educators to create psychologically safe (i.e. the student feels that their relationship with the educator is trustworthy and “safe for interpersonal risk taking,”^19^ ^(p560)^) opportunities as they hold power within the student‐healthcare consumer‐educator triad. Students highlighted the challenges of finding the appropriate time and approach to discuss their questions and concerns, particularly in busy clinical settings. Clear communication from educators to enable approaches to difficult conversations was perceived as crucial for a supportive learning environment.

**TABLE 3:** Sub‐themes, properties of sub‐themes and indicative quotes for the theme “Students need safety to initiate conversations about perceived low‐value care on clinical placement.”

Subtheme	Properties of sub‐theme	Indicative quote/s
Seeking psychologically safe opportunities	Psychological safety enables students to initiate conversations about low‐value care. To facilitate this sense of safety, students need educators who provide a welcoming atmosphere and provide nonjudgmental feedback	“What comes to mind straightaway is [the educator saying] in a one‐on‐one setting that ‘no matter what happens, you could always come to me if you have a question about something, if you are uncomfortable about anything’—even if it is related to that person [the educator]. [Being] reassured that it is okay … not that it is just okay, but they want to hear that feedback.” _[ID01_Radiog]_
Minimizing consequences for the student and consumer	Students assess potential consequences before initiating low‐value care conversations. Students are cautious about raising concerns that might create a conflict with supervisors and negatively impact student learning and assessment. Students are also wary about raising concerns that may make healthcare consumers feel less confident in their care	“We'll be spending six weeks with this supervisor … ultimately, their grades make you or break you. And if in week two, you ruffled some feathers, bad blood is not always worth the risk.” _[ID22_PT]_
		“I'd never ask any of this stuff in front of the patient. You don't want them feeling like there's disagreement within the team and that they're not getting good care.” _[ID10_Para]_
Finding space to approach educators can be tricky	Students need an appropriate space in the clinical environment to initiate conversations about low‐value care. It is challenging for students to find the right time to discuss issues and pick up on cues when the supervisor is busy with other priorities	“If you've got a really busy day, and you've got a lot you need to do, then sometimes having that conversation really close to when the actual situation happened may not even be … feasible …. You kind of miss your opportunity where everything's fresh in your mind.” _[ID19_DT]_
Repeating questions is a flag for reflection	Students need educators who allow repeated questions to enable effective learning. Educators who position repeated questions as inattentiveness rather than a desire to learn do not enable the safety needed for students to initiate conversations around low‐value care	“I understand that it can be quite like irritating to the pharmacist or … anyone really, to explain something … multiple times, but again … the preceptor should keep it in mind that a student is someone with very minimal, if any practice, and they don't necessarily … understand [if] this … same knowledge can be applied to different … situations or not.” _[ID51_Pharm]_

#### Theme 3: Students seek practical training and clear guidance to handle complex conversations during placement

Students felt that incorporating training into academic education and conversing with their educators about low‐value care may benefit them (Table 4). They expressed the need for training beyond generic instructions, underscoring the necessity of specific, scenario‐based guidance. Students highlighted that due to the nuanced nature of these conversations, it was helpful to understand different options, scenarios, and methods for having them.

**TABLE 4:** Sub‐themes, properties of sub‐themes and indicative quotes for the theme “Students seek practical training and clear guidance to handle complex conversations during placement.”

Subtheme	Properties of sub‐theme	Indicative quote/s
Authentic communication training	Enabling students to initiate conversations about low‐value care requires training that aligns with real‐world practice using a practical approach	“Touching on this topic … university will definitely provide guidance on how to start in the first place, especially if students don't have any working experience or if this is their first placement; it might all be very new to them. So it would be good to have a structured approach or some tips on how to talk to someone about … low‐value care.”_[ID49_Pharm]_
Training that enables speaking up safely	Students require clear guidance from education providers on how to raise issues without fearing negative consequences for their academic performance. This includes enabling permission to speak up and framing when and how to speak up	“Before every clinical placement, we have … a lecture that … the unit coordinator will go through … things that we need to do [and] not do: … what to wear, what not to wear … it would be absolutely amazing if they would … say …” of course, you're going to have questions throughout the placement. Do these things if you want to [ask] a question.” Or “if you want to initiate a conversation about … low‐value care, or anything else, … this is the way to go about it. And don't be afraid to go about it. It's not going to affect your degree, it's not going to affect your University markings.” _[ID51_Pharm]_
The university can prepare us for some of this, not all	Although training would enable students to practice initiating conversations about low‐value care in a simulated setting, students recognized the nuanced and situation‐specific nature of conversations in a complex clinical environment could not be fully replicated	“They [the university] tell us … to report any ‘not adequate’ care to … the nurse in charge … They also teach us … how to de‐escalate situations, but that is usually with patients and their visitors and stuff, not really with other nurses … they do tell us to escalate care: “Don't be scared to step up” and all that, but, you know, it is not … always so easy. Especially as a starting student, to escalate care like this that you see.” _[ID40_NM]_

There was general agreement with the clinical educators' recommendations reported by Sevenhuysen et al.^6^ when presented to student participants during the interview. Some students remarked that these recommendations were common‐sense approaches, but knowing they came from educators and seeing them in writing was helpful. Students almost exclusively reported feeling they needed formal training to converse about low‐value care in their academic education. Most students felt their most relevant training was general information about professionalism and communication. Some students reported that their education provider encouraged them to speak up if they saw something, while others reported being told not to question practice at all. Generally, participants felt that they were not adequately prepared to engage in conversations about low‐value care.

Students also debated whether to speak up in certain situations. They recognized that while education providers can prepare them for some aspects, practical experience during WIL placements is crucial for learning how to handle these challenging conversations. The leading training suggestions from students were interactive, including role‐play or simulation, an interdisciplinary group assignment involving reflective writing, or a workshop where supervisors were invited to the education provider to discuss with students how they would like students to bring up these conversations as well as their own experiences of having these conversations with colleagues.

## DISCUSSION

This study identified student health professionals' conceptualisation of low‐value care delivery and their perceptions of educator recommendations about approaching challenging conversations during WIL. Students' definitions of low‐value care were broader than the cost‐and value‐oriented definition of Badgery‐Parker et al.^4^ and extended beyond adherence to clinical guidelines and fiscal indicators to the importance of personal factors such as kindness in care and providing care that engenders trust. This student perspective is consistent with previous studies involving nursing students^10^, ^20^ and healthcare consumers' definitions of low‐value care related to their lived experiences,^21^ indicating that health professional students may be in an ideal position to connect with the needs and preferences of healthcare consumers during WIL placements. This study's findings also suggest that a students' willingness to engage in conversations challenging observed practice during WIL may depend on whether they observe kindness and trust‐building behaviors practiced by their educators when interacting with others in the healthcare setting. Students also expressed a desire for educators to be more explicit about raising questions and to create space during WIL for conversations about the quality of care. These findings point to a potential contradiction in the preferences of students, educators, and healthcare consumers, which has implications for the design of training for students and health professionals involved in clinical education.

Previous research in nursing has found that student nurses were aware of the importance of speaking up if low‐value care was witnessed, but there was a trade‐off between internal beliefs, their understanding of professional standards, and fear of the potential ramifications of speaking up.^20^, ^22^, ^23^ Similarly, this study observed that students from a broader range of health professions described decision‐making processes around speaking up that centered on concern for the patient versus their academic progress. The reluctance to speak up in healthcare settings is not unique to healthcare students. Health professionals report hesitancy to speak up to observed safety concerns with fears of creating conflict, negative responses, and concerns about appearing incompetent’.^24^

Student, health professional, and healthcare consumer perspectives of low‐value care share commonalities. However, differences in the agency of stakeholders in these three groups are an important mediator of the likelihood of conversations challenging practice occurring. Organizational approaches may disempower healthcare consumers; health professionals may need more agency when leaders and superiors do not support speaking up; and students may fear a threat to their assessment outcomes by speaking up. Furthermore, students may have difficulty appreciating the difference between low‐value care that is, and is not, in the best interests of the healthcare consumer. There are times when healthcare legitimately deviates from best practice, for example, to support adherence or build therapeutic alliance,^25^ but this may not be readily appreciated by students during WIL.

One way to support student speaking up behavior during WIL could be to build professional and intentional noticing skills. Professional and intentional noticing is “the capacity to notice and to learn from experience.”^26^ ^(p305)^ Educators are ideally positioned to provide practical guidance to students on applying the skills of noticing and questioning practice in clinical contexts. Through supported questioning behavior, educators can help students understand care contexts and understand why ‘best practice’ may not occur for legitimate reasons in everyday practice. Bringing healthcare consumers into these conversations when appropriate, further extends this idea. This is consistent with reports of healthcare consumer appetite for involvement in discussions with health professionals about their care,^21^ and as providers of feedback on student performance.^27^, ^28^, ^29^ It is acknowledged that action needs to be informed by all stakeholders—healthcare consumer, educator, student‐ to explore and diffuse tensions related to positionality and identify how to build safe spaces for dialogue about healthcare practices.

The work to prepare students for raising questions in the workplace is something that academic educators could begin in the pre‐clinical years. This could be enabled by creating safe environments for students to raise questions in the classroom and by welcoming students to initiate conversations that query observed practices in learning activities such as simulation scenarios and objective structured clinical examinations. However, given the implications of low‐value healthcare, an issue that has a personal impact on healthcare consumers and students and is high stakes for all parties, it also points to a need to bring these diverse stakeholder views together in training design. Making assumptions about how any stakeholder views or experiences low‐value care may impact the utility of training programs. Therefore, it is critical that educators, health professionals, students, and healthcare consumers are brought together to co‐design training programs to maximize the likelihood of developing training that can support educators, students, and healthcare consumers to navigate these potentially high‐stakes conversations about healthcare delivery practice.^30^ It is also important to consider how the outcomes of training might be integrated into the workplace, particularly where an organizational culture and structure that may support speaking up behaviors.^7^, ^8^

The WIL context will influence the design of training to make the process of conversations about low‐value care explicit for clinical educators, students, and healthcare consumers. How these conversations can be supported will differ depending on the WIL setting. For example, in high‐acuity settings, the timing and conduct of these conversations may vary from those in primary care. This highlights the role of the educator in orienting students to the local professional culture related to care delivery conversations.^31^ In our study, students indicated that explicit instructions from educators about navigating the process of initiating conversations would be helpful. Students identified factors that they thought would need to be negotiated when moving from context to context in response to our summary diagram of educator's prior recommendations. Students identified the critical role educators can play in setting the tone for negotiating time and space to have conversations about practice and the reluctance of students to initiate these conversations if they had concerns for their own or a patient's safety. The role of educators in promoting psychological safety during WIL has been examined in the context of feedback practices.^19^ Students in the current study expressed a desire for educators to be more explicit about how they should raise questions about care delivery and make space for conversations about the quality of care that was educator‐initiated.

There is little guidance about best practice training interventions for speaking up behavior in healthcare.^32^ Strategies that enable students to approach educators when they observe clinical practice that differs from what they have learned have value, supported by the findings of this study and research from the broader healthcare safety literature.^33^ Making explicit the factors that can negatively impact the willingness of healthcare students or professionals to speak up or reduce the opportunity for healthcare consumers to be heard may be an important part of a training program. By understanding these factors from all stakeholder perspectives, we can enhance training for students and educators to facilitate the development of their speaking‐up behavior as an integral part of their professional identity. Given the complexity of navigating these conversations, a staged approach to training is likely needed, starting with pre‐clinical education providers, moving from theoretical to simulation‐based training methods, which could include educators, and then moving to in‐situ practice while on work‐integrated learning placements. Universities could consider revising or expanding their curricula to address the difficulties in raising questions about an educator's practice and the approaches revealed within the findings. These learning opportunities in the pre‐clinical curriculum could support students in questioning their educators' practices appropriately. It is important to note that similar to the dialogue about feedback conversations in health professions education,^19^ educators need to develop and practice psychological safety when supporting students in navigating conversations about low‐value care.

### Strengths and limitations

The strength of this study was the use of team‐focused approaches to data collection and analysis procedures. Research team members were from various health professional, educational, and research backgrounds, and multiple perspectives could be drawn on throughout the project to maximize reflexivity and rigor. A potential limitation of this study is that, although the overall broad representation of student disciplines and experiences within the data aids the transferability of findings, the degree of representation across each profession varied, and despite recruitment efforts, some disciplines could not be recruited. It should also be noted that although trans‐Tasman recruitment was attempted, most participants were from the Australian State of Victoria.

## CONCLUSIONS

This study highlights that health professional students perceive low‐value care as a multifaceted issue beyond adherence to clinical guidelines, encompassing respect, compassion, and trust in care delivery. Students identified a need for safety and explicit guidance from educators to navigate conversations about low‐value care during WIL. Incorporating structured, scenario‐based training focusing on raising low‐value care issues into academic curricula and fostering psychologically safe environments during WIL placements may enable students to approach these conversations confidently. These findings suggest co‐designed educational interventions involving students, educators, and healthcare consumers to enhance communication skills about low‐value care warrant exploration.

## AUTHOR CONTRIBUTIONS

**Melanie K. Farlie:** Conceptualization; investigation; funding acquisition; writing – original draft; methodology; validation; visualization; writing – review and editing; formal analysis; project administration; data curation; supervision; resources. **Jessica Coventry:** Writing – original draft; validation; writing – review and editing; formal analysis; project administration; investigation; methodology; visualization; data curation. **Jonathan Foo:** Conceptualization; investigation; funding acquisition; writing – original draft; validation; writing – review and editing; formal analysis; methodology. **Samantha Sevenhuysen:** Conceptualization; funding acquisition; writing – original draft; methodology; validation; writing – review and editing; formal analysis. **Brendan Shannon:** Investigation; writing – original draft; validation; writing – review and editing; formal analysis; methodology; conceptualization. **Cylie M. Williams:** Conceptualization; funding acquisition; writing – original draft; validation; writing – review and editing; formal analysis; methodology. **Stephen Maloney:** Conceptualization; funding acquisition; writing – original draft; methodology; validation; formal analysis; resources. **Kristie Matthews:** Conceptualization; investigation; writing – original draft; validation; writing – review and editing; formal analysis; project administration; data curation; supervision; methodology.

## FUNDING INFORMATION

This research was funded in part by an Australian and New Zealand Association of Health Professional Educators Research Grant 2022.

## CONFLICT OF INTEREST STATEMENT

The authors declare no conflict of interest concerning this publication.

## ETHICS APPROVAL

This research was approved by the Monash University Human Research Ethics Committee #35565.

## Supporting information


Data S1.
