# Supplementary material for: “We might be put into situations we are uncomfortable with, but not exactly told how to deal with them”: Health professional students' experiences questioning low‐value care practices during work‐integrated learning
Source: Anat Sci Educ. 2025 Jun 5;19(2):190–200. doi: 10.1002/ase.70054 (PMC12934367; doi:10.1002/ase.70054)
Supplement: Supplementary file 1 — Data S1. [file ASE-19-190-s001.pdf]

Supplementary material file

Appendix 1a - Interview guide

<u>Opening</u>

Thank you for agreeing to participate in this interview today.

Today, I would like to hear about your experiences initiating conversations on clinical practice/placement about what you perceived as 'low-value care'. I value your honest opinions, and because this interview is mainly unstructured, you can contribute any discussion points that you believe are important to this topic. Where possible, please refer to any peers or colleagues by their first name only, and try not to refer to your clinical placement service by name. The interview will be audio and video recorded for data analysis. If you want to stop recording this interview at any time, please raise your hand, and I will stop the recording.

Do you have any questions before we start? I will now start the recording.

**Questions**	**Focus**	**Relevant Prompts**
1. Please tell me about your clinical placement experiences so far. What have they been like?	Baseline experiences	Type of service/s<br>Duration<br>On placement as a solo student or with peers<br>Supervision and support structure
2. This research focuses on low-value care. How do you understand low-value care in your profession?	Perception of meaning<br>Context of meaning	
3. Could you tell me about any experiences you had observing low-value care [or their preferred term] on your placement? How did you respond?<br><br>If you've not observed low-value care [or their preferred term] on your placement, how might you respond if you observed low-value care?	Conversation action/s	
4. How willing were you/would you be to have a conversation discussing your observations of low-value care [or their preferred term] while on a clinical placement?	Conversation confidence	

5. What influences your willingness to talk about low-value care [or their preferred term] seen on clinical placement?	Conversation influencing factors	Barriers and enablers to initiating conversations, Perceived student role<br>Influencing factors to initiate conversations – i.e. skills, prior training, prior experience, confidence, culture, empowering others, the role of the professional/s being observed delivering that care<br>Did you feel adequately trained to engage in these conversations, and if not, do they feel it is something they could be trained for?
6. What do you perceive the clinical educator/tutor/supervisor's influence in your decisions to discuss low-value care [or their preferred term] while on placement?	Role of influential others	Guidance on how, when, where, and why to act<br>Empowering or limiting action
7. We completed a prior study exploring clinical educator perceptions of where, when, and how students should initiate conversations after observing low-value care [or their preferred term]. I want to share a graphic showing their responses with you now. What do you think about these educator perceptions?<br>Use of visual aid	Conversation context<br>Barriers and enablers	Alignment with student perceptions – similarities and differences, reasons why<br>Barriers and enablers to achieving educator context

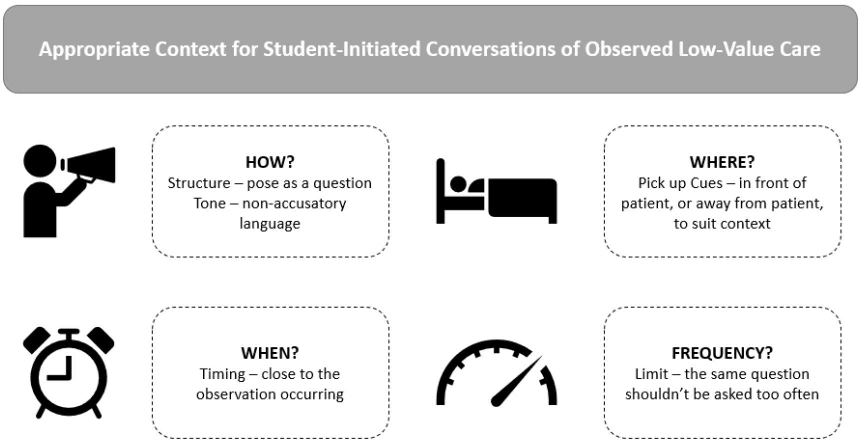

8. Is there anything you would like to add related to this topic that we have not discussed?		

Appendix 1b – Interviewer post-interview debriefing form

Example of a completed structured post-interview debriefing form used by and shared between all interviewers in this study to guide personal reflexivity and the team's approach to each subsequent interview

1. What main issues or themes emerged from this interview?

Passionate about ethical care. A mature-aged student has a family that is also a health professional. Has an active interest in the topic but was unclear on the topic definition? They had not read the BMJ article included in the explanatory statement.

2. What new information did you gain through this interview?

Different mature-age student perspective. Less brazen about speaking out compared with [prior interviewee] - this interviewee self-reflected that 'speaking out' and being 'too direct' and misunderstood had been issues in the past, so cautious about challenging supervisors on placement

3. What new questions emerged for you due to this interview?

I want to ask more about how prior experiences influence actions as a student.

4. What else was salient, interesting, illuminating, curious or important during this interview?

Thoughtful and very invested in the interview topic. There was a conversation about the ethics of billing practices in private radiology, but this was something that the student did not challenge as the clinicians did not either - everyone wants to get their job done - and needs to choose their battles -but the student appeared uncomfortable when speaking about the inequity of access. We also covered the impact of non-inclusive language and the practice of clinicians impacting willingness to speak up.

5. What new or remaining concerns, inconsistencies, or challenges emerged during this interview?

I want to ask more about how prior experiences influence actions as a student, but it is hard at times to weave in without straying too far off-topic.

Looking for ways to guide the interviewee to talk about seeing low-value care, even if they have not recognised it as such, without leading them in the interview

6. What has been further confirmed or challenged in this interview compared with previous interviews?

Speaking up can be misinterpreted; even as a mature student, this may not change the willingness to speak out. Early in training, students may not be as aware of what low-value care is or looks like and may not be able to process it even if it is seen.

7. How would you describe the general attitude of the interviewee towards the topics discussed?

They are very invested in and interested in the topic. They have a strong moral compass, but they are aware that they may choose to stay quiet rather than speak up if they feel their assessment or supervisor relationship may be impacted by speaking up.

8. Overall, how would you describe the interviewee's participation in the interview?

engaged and articulate

9. What did you observe that would not be evident from reading a transcript (e.g. individual behaviour etc.?)

sitting outdoors, relaxed, thoughtful

10. What problems did you encounter (e.g., logistical, behaviours of the individual, confusing questions, etc.)?

Some wind, at times, may affect the clarity of the recording. Add a prompt to the invite to be in a quiet, private indoor space (if possible) for the interview.

Appendix 2 - Team Reflexivity syntheses at project initiation, pre-recruitment and data analysis stages.

Reflexivity statements from individual team members were collected using an online survey using reflective prompts adapted from Barry, C.A. et al. (1999). Using reflexivity to optimise teamwork in qualitative research. *Qualitative Health Research*, 9(1), 26-44.

**Project stage** / **Theme**	**Project initiation**	**Pre-recruitment**	**Data analysis stage**
**Experience with student learning influences our perspectives.**	All researchers are experienced in student clinical education (as delivering education or on the receiving end as a student), and prior views may influence approaches and pre-conceptions towards study findings, particularly barriers and enablers to student learning on clinical placement. We expect that students will be able to identify low-value care they have observed during WIL but are open to exploring what students perceive as low-value care. The team is motivated by a desire to ensure students are optimally supported during WIL to speak up about practices they observe and perceive as low-value care.	The team's reflections highlight how past experiences with student learning continue to shape their perspectives on this project. The team anticipate finding that while students may be taught about evidence-based practice and professional dialogue, they may lack structured opportunities to apply these skills in real-world clinical settings. Past experiences suggest that students hesitate to question authority figures in clinical settings.	Similar to the project initiation reflections, all researchers acknowledged that their experience in student clinical education may influence their approaches and interpretation. Two members acknowledged that their teaching/educator role may lead to a bias towards believing that the skill set required to have conversations about LVC is a skill set that can be taught.
**Students are unlikely to raise their voices if they observe what they perceive to be low-value care.**	Students are likely to be disempowered on placement and must be brave and not afraid to respond to observations of low-value care. Interestingly, the student’s capacity to observe low-value care is not questioned. This also aligns with the perceived project risks that students may not make visible placement-related challenges if they feel a power imbalance between placement supervisors or academic researchers.	The team expects low engagement in challenging conversations, not necessarily due to a lack of knowledge but rather due to power dynamics, fear of repercussions, and professional socialisation. This expectation stems from direct experience with students who have struggled to assert themselves when encountering low-value care, reinforcing the need to explore how power relations influence student behaviour.	The team reflected that this project's findings are more diverse and nuanced than anticipated. Many students report speaking up, but cultural norms, power, confidence, and knowledge can disempower them. The project intent was to explore training for students. Given the contextual and cultural challenges in the workplace, it's difficult to visualise what this might look like in the absence of workplace training.
**Collecting student perspectives**	As a team, we anticipate seeing a spectrum of bravery reported by students about speaking up on placements. We anticipate that the students'	Concerns about student recruitment and their ability to identify low-value care also reflect prior experiences in student learning. The team is	The students we interviewed provided definitions of low-value care broader than cost and value considerations. These definitions were wider than

**on low-value care is critical to understanding how to support students during clinical education**	perspective on managing tricky conversations is challenging, and they may not feel empowered to have a voice when observing substandard behaviour. We anticipate the enculturation of the workplace to be a feature where behaviour observed on placement may take precedence over what is learned at University.	aware that students may not always have the clinical expertise or confidence to recognise when care is suboptimal and that even when they do, they may lack the skills or perceived authority to challenge it. The team acknowledges that students may overstate their confidence or willingness to engage in these discussions due to pressures related to social desirability. These reflections highlight the importance of conducting interviews that account for students' developmental stages and psychological safety.	anticipated and surprisingly reassuring to some research team members. Other team members were also saddened and disappointed by the low-value care observations reported in the study.
**Analytical lenses**	Half the team identified social interactionist theory with a critical lens and their primary stance; however, half did not have a stance. We may need to strengthen this when it comes to analysis.	The team continues to have members with and without stances on a theoretical lens. Those without a strong stance are interested to learn more about these from the broader team. Those with a stance currently see this study as situated within an interpretivist paradigm as the research prepares to capture students' subjective experiences and explore broader institutional, social, and cultural influences on those experiences.	Half the team took a stance on lenses and orientations. While critical inquiry was initially perceived as the primary relevance of this work, the team now considers that other lenses, such as a social constructionist lens, are needed to interpret the nature of the data in this study.
**Motivation to empower student voices drives our curiosity**	A collective desire to use the research as a tool to empower voice on placement through staff intervention or student learning opportunities	The team's desire to produce practical, evidence-based guidance for students and educators is grounded in its collective experience working with students who struggle to navigate difficult conversations. The team's concerns about workload, project completion, and dissemination also stem from an understanding that meaningful change in education takes time and resources, reinforcing the need for well-supported implementation of findings.	Reflections echo those in the project initiation and pre-recruitment stages with the research team's shared goal of providing practical strategies for staff and students to encourage more conversations about low-value care.

Appendix 3—Participant and Interviewer discipline and transcript allocations for thematic framework construction by discipline. The role of the coder is indicated as either an interviewer or a non-interviewer member of the research team. *denotes that the allocated coder also interviewed that participant.

			Round 1		Round 2	
			Coder 1	Coder 2	Coder 1	Coder 2
ID	Participant discipline	Interviewer discipline	Coder Discipline (project role)			
13	Dietetics	Physiotherapy	Physiotherapy (non-interviewer)	Physiotherapy (interviewer*)		
14	Dietetics	Physiotherapy				
19	Dietetics	Physiotherapy			Podiatry (non-interviewer)	Paramedicine (interviewer)
40	Nursing/Midwifery	Physiotherapy	Podiatry (non-interviewer)	Radiation Therapy (interviewer)	Physiotherapy (non-interviewer)	Physiotherapy (non-interviewer)
42	Nursing/Midwifery	Physiotherapy				
29	Occupational Therapy	Physiotherapy	Physiotherapy (interviewer)	Physiotherapy (non-interviewer)		
30	Occupational Therapy	Radiation Therapy				
31	Occupational Therapy	Physiotherapy				
33	Occupational Therapy	Physiotherapy				
36	Occupational Therapy	Physiotherapy			Paramedicine (interviewer)	Physiotherapy (non-interviewer)
10	Paramedicine	Physiotherapy	Physiotherapy (interviewer)	Paramedicine (interviewer)		
11	Paramedicine	Radiation Therapy			Paramedicine (interviewer)	Physiotherapy (non-interviewer)
45	Pharmacy	Physiotherapy				
47	Pharmacy	Physiotherapy				
49	Pharmacy	Physiotherapy	Radiation Therapy (interviewer)	Physiotherapy (non-interviewer)		
51	Pharmacy	Physiotherapy				
53	Pharmacy	Radiation Therapy			Physiotherapy (interviewer)	Physiotherapy (interviewer)
07	Physiotherapy	Radiation Therapy	Physiotherapy (non-interviewer)	Paramedicine (interviewer)	Podiatry (non-interviewer)	Radiation Therapy (interviewer*)

Continued…

Continued…

				Round 1	Round 2	
			**Coder 1**	**Coder 2**	**Coder 1**	**Coder 2**
**ID**	**Participant discipline**	**Interviewer discipline**	**Coder Discipline (project role)**			
08	Physiotherapy	Radiation Therapy				
18	Physiotherapy	Paramedicine				
22	Physiotherapy	Radiation Therapy				
28	Physiotherapy	Paramedicine				
35	Podiatry	Physiotherapy	Physiotherapy (non-interviewer)	Podiatry (non-interviewer)	Podiatry (non-interviewer)	Radiation Therapy (interviewer)
24	Radiation Therapy	Physiotherapy	Paramedicine (interviewer)	Radiation Therapy (interviewer)		
44	Radiation Therapy	Physiotherapy				
46	Radiation Therapy	Physiotherapy				
01	Radiography	Physiotherapy				
02	Radiography	Physiotherapy	Podiatry (non-interviewer)	Physiotherapy (interviewer)	Physiotherapy (non-interviewer)	Physiotherapy (non-interviewer)
03	Radiography	Physiotherapy				
04	Radiography	Physiotherapy				
05	Radiography	Physiotherapy				
06	Radiography	Paramedicine				
16	Radiography	Physiotherapy				
17	Radiography	Radiation Therapy				
23	Radiography	Physiotherapy				
38	Social Work	Physiotherapy	Physiotherapy (interviewer)	Physiotherapy (non-interviewer)	Radiation Therapy (interviewer)	Physiotherapy (interviewer)
